# Interaction of a Histidine-Rich Antimicrobial Saliva Peptide with Model Cell Membranes: The Role of Histidines

**DOI:** 10.1021/acs.langmuir.3c00498

**Published:** 2023-05-25

**Authors:** Amanda E. Skog, Giacomo Corucci, Mark D. Tully, Giovanna Fragneto, Yuri Gerelli, Marie Skepö

**Affiliations:** † Division of Theoretical Chemistry, Department of Chemistry, Lund University, P.O. Box 124, SE-221 00, Lund, Sweden; ‡ Institut Laue-Langevin, 71 avenue des Martyrs, 38000, Grenoble, France; § BM29 BIOSAXS, European Synchroton Radiation Facility, 71 avenue des Martyrs, Grenoble, Isère 38043, France; ∥ European Spallation Source ERIC, P.O. Box 176, SE-221 00 Lund, Sweden; ⊥ CNR Institute for Complex Systems, Uos Sapienza, Piazzale Aldo Moro 2, 00185 Roma, Italy; # Department of Physics, Sapienza University of Rome, Piazzale Aldo Moro 2, 00185 Roma, Italy; ∇ LINXS - Institute of Advanced Neutron and X-ray Science, Scheelevägen 19, SE-233 70, Lund, Sweden

## Abstract

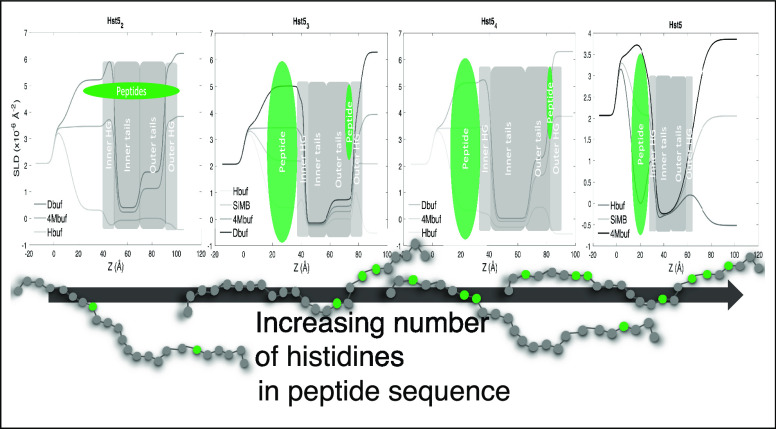

Histatin 5 is a histidine-rich, intrinsically disordered, multifunctional saliva protein known to act as a first line of defense against oral candidiasis caused by *Candida albicans*. An earlier study showed that, upon interaction with a common model bilayer, a protein cushion spontaneously forms underneath the bilayer. Our hypothesis is that this effect is of electrostatic origin and that the observed behavior is due to proton charge fluctuations of the histidines, promoting attractive electrostatic interactions between the positively charged proteins and the anionic surfaces, with concomitant counterion release. Here we are investigating the role of the histidines in more detail by defining a library of variants of the peptide, where the former have been replaced by the pH-insensitive amino acid glutamine. By using experimental techniques such as circular dichroism, small angle X-ray scattering, quartz crystal microbalance with dissipation monitoring, and neutron reflectometry, it was determined that changing the number of histidines in the peptide sequence did not affect the structure of the peptide dissolved in solution. However, it was shown to affect the penetration depth of the peptide into the bilayer, where all variants except the one with zero histidines were found below the bilayer. A decrease in the number of histidine from the original seven to zero decreases the ability of the peptide to penetrate the bilayer, and the peptide is then also found residing within the bilayer. We hypothesize that this is due to the ability of the histidines to charge titrate, which charges up the peptide, and enables it to penetrate and translocate through the lipid bilayer.

## 1 Introduction

The development of antibiotic resistance is a serious and growing public health problem, representing today one of the biggest threats to world health and development, according to the World Health Organization. Therefore, finding alternative ways to prevent and treat infectious diseases is paramount. Antimicrobial peptides (AMPs) have received considerable attention as an alternative to traditional antibiotics as they are a natural part of our innate immune system and a possible alternative to conventional antibiotics. Most are of natural origin and are part of the innate immune response found among all life classes. Bacteria have coexisted and coevolved with AMPs without developing significant resistance.^1^ These molecules are unique and diverse and can be divided into subgroups based on their amino acid composition and structure; for example, they can be classified into four groups: extended AMPs, β-hairpin or loops, β-sheet, and amphipathic α-helical.^2,3^ Many of these peptides are intrinsically disordered in solution and fold into their final configuration upon partitioning into biological membranes.^2,4^

In this study, we focus on extended AMPs, which are characterized by sequences rich in glycine (Gly), arginine (Arg), and histidines (His), containing little or no secondary structure.^2^ We use the antibacterial and antifungal saliva peptide Histatin 5 (Hst5) as a model peptide. Hst5 is a 24 amino acid long, intrinsically disordered,^5^ multifunctional, cationic peptide,^6^ known to have antimicrobial properties and to act as the first line of defense against oral candidiasis caused by *Candida albicans*.^7^ For this fact alone, it is an important peptide to study since *Candida albicans* is responsible for roughly 70% of all global fungal infections, including periodontal disease.^8^ The Histatin family consists of 12 members, where Hst5 is the most potent concerning antibacterial and antifungal activity,^9,10^ which has been ascribed to the high content of basic amino acids. The ionic strength has also been reported to play a role,^11^ which is relevant to consider since the ionic strength in saliva can range from 30 mM to 100 mM.^12^ Hst5 behaves as a random coil under physiological conditions,^13−15^ although it possesses some degree of polyproline II helical structure.^16,17^ It has been found in several studies that the peptide attains an α-helical structure in non-aqueous solvents such as 2,2,2-trifluoroethanol (TFE)^14,18,19^ but also in the vicinity of lipid vesicles.^20^ Since 7 of 24 amino acids are His (≈ 30%), where the conjugated imidazole side chain has a p*K*_a_ value of approximately 6, one can expect the electrostatic mechanisms of charge titration to play an important role. We have shown this in simulations,^21−23^ but, so far, without confirming it experimentally.

It has been reported in several previous studies that the antimicrobial effect of Hst5 does not follow the typical route of killing through pore formation in the microbial cell membrane.^18,19,24,25^ Instead, Hst5 translocates across the cell membrane, accumulating intracellularly in the mitochondrion.^25−27^ For this to happen, it is crucial that there is a membrane potential on the mitochondrion;^11^ hence, a negative charge inside the plasma membrane is needed for Hst5 to kill the target microbe. Due to this observation, it has been suggested that Hst5 is a cell-penetrating peptide (CPP) and thus a peptide that is able to translocate lipid membranes without disrupting the membranes’ integrity. The translocation of Hst5 *in vivo* occurs without active transporters and only induces limited, temporary damage to the plasma membrane.^24,26^

**Figure 1:**
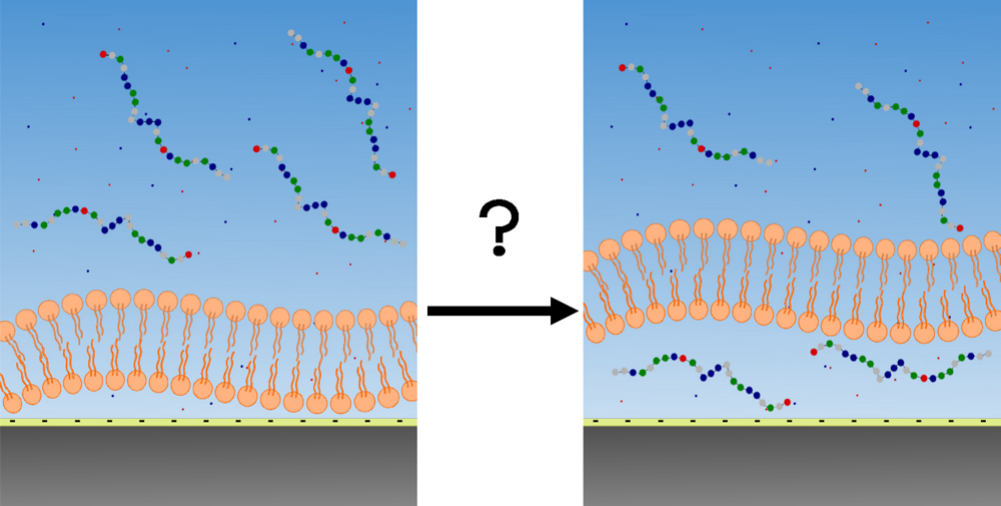
Schematic representation (not to scale) of Hst5 above and below supported lipid bilayers forming a cushion. Reproduced or adapted from Gerelli et al.^30^ Copyright 2020 American Chemical Society.

The fact that Hst5 contains a large fraction of His has puzzled us and made us question why and what their role is. For example, it is well-known that Hst5 contains a HExxH zinc motif,^28^ and in a recent study, we have shown that Hst5 contains a possible zinc motif in the N-terminal as well, HAKRHH.^29^ Under physiological conditions, these two motifs bind zinc and induce a dynamic oligomer formation. To understand the role of the His on the interaction with cell membranes and the role of electrostatic interactions, the measurements were performed at low ionic strength (10 mM). It has been shown in a previous study by us that at low ionic strength, Hst5 translocates across a charged bilayer and lifts it from a solid surface without disrupting the internal structure of the bilayer. Hence, a cushion is formed.^30^ A schematic of this is shown in Figure 1.

The working hypothesis for the mechanism of action is the following: the driving force for the adsorption of an oppositely charged macromolecule to a solid surface has an entropic origin stemming from counterion release. When a lipid bilayer is deposited on a solid surface, such as silica in our case, the system consists of one solid surface and two fluidic interfaces with accompanying counterions. The pH and the ionic strength can be considered equal on both sides of the bilayer since bilayers are known to be permeable to ions and other small molecules. In contrast, the dielectric constant can differ in the substrate-solid supported lipid bilayers and bulk. Such an effect has recently been demonstrated for like-charged interacting surfaces using experiments^31,32^ and computer simulations.^33^ Hence, when a peptide such as Hst5, which can charge titrate due to its high content of His, approaches the head groups of the lipid bilayer facing the bulk solution, it will slightly increase its charge as proven by computer simulations by our group and diffuse through the bilayer.^21−23^ According to the simulations, the length scales when protonation is initiated and the thickness of the bilayer are of the same approximate order of magnitude. Hence, the peptide recognizes the electrostatic field from the inner bilayer head groups and the solid silica surface while already in the bulk solution. Adsorption to the silica surface is most likely promoted by counterion release, which induces an increase in osmotic pressure between the silica surface and the bilayer, thereby forming a cushion. Hence, this means that Hst5 has crossed the model cell membrane. The reader should notice that no Hst5 translocation across the lipid bilayer nor cushion formation has been observed when the solid silica substrate was replaced by sapphire.^30^

To respond to the hypothesis that the charge titration mechanism could be important for histidine-rich AMPs and, more specifically, our model peptide Hst5, four variants have been used where the original seven His have been reduced to four, three, two, or zero, respectively.^29^ The sequences of all peptides are presented in Table 1. In this study, we have been focusing on three different conditions of the peptides:

1. Bulk solution, to evaluate if the designed peptides behave similarly to the wild-type Hst5.
2. Interaction with a solid surface, a silica surface, or a silica particle. This is performed to understand the interaction with the solid substrate, which will be present in our supported lipid bilayer (SLB) system, to consider if this affects the results we observe for that system.
3. Interaction with a SLB consisting of a mixture of negatively charged and zwitterionic phospholipids deposited on a solid silica surface. This is performed to investigate the ability of the peptides to translocate the SLB when the number of His is varied, as well as to understand the adsorption properties. Here, the solid silica surface acts as a model to mimic the mitochondrial potential in a cell.

**Table 1:**
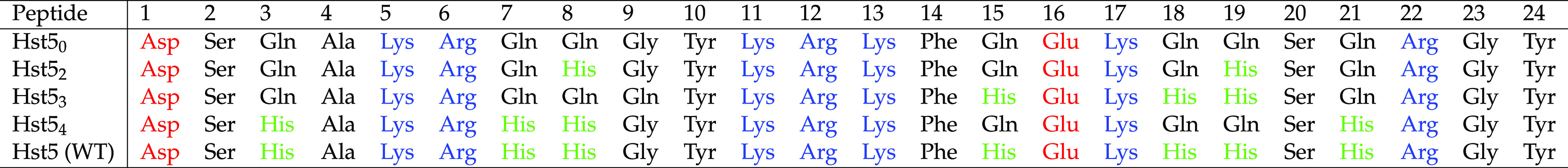
Sequences of the Peptide Variants Investigated in the Present Work^a^

a Positively charged amino acids are presented in blue, negatively charged in red, and histidines in green.

Upon replacing the number of histidines in Hst5 by their pH-insensitive counterpart glutamine (Gln) we noticed different effects depending on the peptide environments. For (1), only minor changes in the peptide conformation and structure were observed by utilizing small angle X-ray scattering (SAXS) and circular dichroism/synchrotron radiation circular dichroism (CD/SRCD). In contrast, upon interaction with a silica surface, as in (2), no structural effects were observed by SRCD. In comparison, quartz crystal microbalance with dissipation monitoring (QCM-D) showed that the adsorbed amount and adsorption processes depended on the number of His. In case (3), we found, by using QCM-D and neutron reflectometry (NR), that the number of His is vital for the penetration depth of the peptide into the bilayer, which is key in the mechanism behind the cushion formation, but also that it gives rise to different adsorption processes.

Moreover, the cushion’s size depends on the conformation of the adsorbed peptide. Hst5, which has a smeared polyelectrolytic distribution of positive charges along the primary sequence, gives rise to the smallest gap compared to the variants with fewer His. We attribute this to flatter adsorption; for example, articles by Kurut et al. and Hyltegren et al.^21−23^ Hence, the number of His is important upon interaction with a lipid bilayer, and most likely also their position in the primary sequence, which is an ongoing study.

## 2 Experimental Section

### 2.1 Peptide Solutions

Hst5 and variants, Hst5_#His_ in Table 1, were purchased from TAG Copenhagen A/S, Denmark, with a purity of 99 and 95%, respectively, determined by high-performance liquid chromatography (HPLC). Before use, the peptides were further purified by dialysis (100–500 Da MWCO Biotech Cellulose Ester (CE) Dialysis Membrane Tubing, SpectrumLabs, Piraeus, Greece) against Milli-Q water at 6–9 °C and lyophilized. Finally, the peptide powder was dissolved in buffer as described later for each experiment.

### 2.2 Vesicle Preparation

Lyophilized 1-palmitoyl-2-oleoyl-*sn*-glycero-3-phosphocholine (POPC) and 1-palmitoyl-2-oleoyl-*sn*-glycero-3-phospho-l-serine (POPS) were purchased from Avanti Polar Lipids (Alabaster, USA). Stock solutions were prepared in a 3:7 methanol:chloroform mixture using the lipid molar ratio POPC:POPS 9:1 (PC_POPC_:PS_POPS_ in the text).

The methanol:chloroform mixture was evaporated under nitrogen flow to form a lipid film, and any remaining solvent was evaporated under reduced pressure (0.8 bar) overnight. The lipid films were hydrated in 500 mM NaCl, 20 mM Tris buffer at pH 7.4. Large unilamellar vesicles (LUVs, 100 nm nominal size) were obtained by extruding the lipid suspensions 31 times through a 0.1 μm polycarbonate membrane filter (Avanti Polar Lipids, Alabaster, USA).

### 2.3 Vesicle Fusion Protocol

To obtain SLBs, the vesicle fusion^34,35^ protocol optimized in ref (30) was utilized. In brief, the injection of LUVs was performed in 500 mM NaCl buffer, and the vesicles were left to incubate for 60 min, followed by a rinsing step with 10 mM NaCl buffer (20 mM Tris at pH 7.4) to induce osmotic shock. This resulted in reproducible, high-quality PC_9_PS_1_ bilayers.

### 2.4 Quartz-Crystal Microbalance with Dissipation Monitoring

QCM-D is a powerful technique that can be used to monitor adsorption processes and changes in the viscoelastic properties of films at solid interfaces.^36^ Briefly, this technology exploits the measurement of changes in the vibrational oscillations of a quartz crystal when an alternating potential is applied to it. Changes in the frequency (Δ*F*) and dissipation (Δ*D*) of the oscillation can be related to the mass associated with the deposited material and its viscoelastic properties. QCM-D data were analyzed by evaluating the trends of the normalized frequency shifts (, where *n* is the overtone number and Δ*F*_*n*_ is the frequency response at the *n*th overtone) and of the dissipation factors (Δ*D*_*n*_). In the case of rigid thin films, the Sauerbrey equation can be used to evaluate changes in adsorbed mass per unit area, Δ*m*, as^37,38^
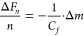
1where *C*_*f*_ is the mass sensitivity constant (*C*_*f*_ = 17.7 ng cm^–2^ Hz^–1^ for an AT-cut quartz crystal with 5 MHz fundamental frequency). The relative uncertainty on the calculated Δ*m* was determined by taking the standard deviation of the QCM-D data in the time interval of interest as a measure of the experimental accuracy. For all samples, it resulted to be in the range 0.5–1.0%.

To gain insight into the peptides’ adsorption behavior and characterize their interaction with silica and SLB surfaces, QCM-D measurements were performed on an E4 apparatus (Biolin Scientific, Sweden) equipped with four thermally controlled flow modules. All experiments were conducted on SiO_2_-coated AT-cut 5 MHz quartz sensors (Biolin Scientific, Sweden). Before usage, the sensors were cleaned by bath sonication first in chloroform, then in acetone, and finally in ethanol (15 min for each solvent). To remove any remaining contaminants and to make the surfaces hydrophilic, the surface was then treated, before use, by air plasma for 3 min (model PDC-3XG, Harrick Plasma, Ithaca, USA). The cleaned sensors were enclosed in the dry flow modules. Before measurements, the flow modules were filled with the same buffer as the sample was dispersed in (20 mM Tris pH 7.4, 10/500 mM NaCl depending on whether measurements were performed on bare SiO_2_ or with an SLB) using a peristaltic pump (Ismatec IPC-N 4, Switzerland) at 0.120 mL min^–1^. The desired solution was injected at a constant flow rate when a stable baseline in an adsorbate-free buffer was obtained. SLBs were formed according to the vesicle fusion protocol described above. A vesicle-containing solution was injected (0.2 mg mL^–1^) at 0.120 mL min^–1^. Once the bilayer was formed, peptide-containing solutions (1 mg mL^–1^) in buffer (10 mM NaCl, 20 mM Tris, pH 7.4) were injected in the cells and incubated for ∼1 h. The samples were then rinsed with the buffer until the frequencies stabilized. For the measurements on bare SiO_2_ the steps to form SLB were excluded from the protocol. Data were collected continuously during all of the steps, and the temperature was kept constant at 20 °C. To ensure reproducibility, 2–4 replicas of the same experiment were performed. The results presented, as well as the QCM-D data reported in the manuscript, are always the average of all replicas performed.

### 2.5 Neutron Reflectometry

Specular NR was used to obtain information on the thickness and composition of the samples, and in particular on the position of the peptides with respect to an SLB and its supporting silica surface, and on the structural modifications induced by the peptides on already formed SLBs. Generally, reflectivity is measured as the ratio between the intensity of the reflected and incident beams, and it is expressed as *R*(*Q*_*z*_), i.e., a function of the scattering vector *Q*_*z*_. In the case of specular reflection, the latter is fully oriented along the direction perpendicular to the sample surface (for convention named *z*), and it can be calculated as
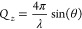
2where λ is the neutron wavelength and θ stands in for both incident and reflection angles.

NR experiments were performed using silicon single crystals as solid substrates (8 × 5 × 1.5 cm^3^, cut along the 111 plane, polished with 3 Å root-mean-square (RMS) roughness, purchased from Sil’tronix ST, Archamps, France). The cleaning procedure was the same as that for QCM-D experiments, except that the substrates were exposed to air plasma for 2 min. After cleaning, the substrates were assembled into water-filled solid/liquid cells provided by Institut Laue-Langevin (ILL; Grenoble, France). The cells were composed of a water reservoir equipped with inlet and outlet valves, allowing the exchange of aqueous solution and injection of peptide solution. This controlled solution exchange is also required to apply the contrast variation method^39^ and was performed using an HPLC pump.

NR measurements were performed on FIGARO,^40^ a time-of-flight horizontal-surface reflectometer at ILL. During our experiments, the instrument was configured to operate with incident wavelengths ranging from 2 to 30 Å and two angles of incidence, namely, 0.62 and 3.8°, resulting in a *Q* range from 0.0045 to 0.42 Å^–1^. To exploit the contrast variation method, measurements were performed mixing, at different ratios, D- and H-buffers (D_2_O- and H_2_O-based, respectively): 100% D-buffer, 100% H-buffer, and a 38:62 D/H-buffer mixture referred to as silicon-matched buffer (SiMB) with an SLD value matching that of crystalline silicon and a 66:34 D/H-buffer mixture named 4MBuffer, with an SLD value of 4 × 10^–6^ Å^–2^. Raw data were converted to reflectivity curves using the COSMOS routine.^41^ The silicon substrates were characterized in both 100% D-buffer and 100% H-buffer before injection of LUVs, which were injected at a concentration of 0.2 mg mL^–1^. After incubation of 1 h and subsequent rinsing steps, the peptides were injected at a concentration of 1 mg mL^–1^.

Information about the samples was derived according to a common slab model using the software application Aurore.^42^ The model consisted of a series of layers, each described in terms of SLD, layer thickness *t*, buffer volume fraction *f*_*w*_, and interfacial roughness σ. The model for the bare substrate consisted of an infinite layer with the SLD of the crystalline silicon, an oxide layer, and an infinite bulk aqueous layer. Upon addition of an SLB, an additional five layers were included to describe the water gap between the solid substrate and the bilayer, followed by the headgroups and tail region of the inner leaflet facing the solid substrate, as well as the leaflet in the proximity of the aqueous bulk face. A schematic figure of this model is available in the paper by Gerelli.^43^ Different scenarios were evaluated for the data obtained after peptide incubation to determine the most suitable model. It was found that it was not necessary to increase the number of layers in the model; indeed, data could be analyzed simply by allowing changes in the SLD values of the existing layers to account for the presence of peptide molecules. The total SLD of a layer composed of *N* chemical species can be calculated as
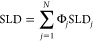
3where Φ_*j*_ (∑_*j*=1_^*N*^ Φ_*j*_ ≡ 1) is the volume fraction and SLD_*j*_ is the SLD of the *j*th molecular species in a given layer. The presence of hydration water was directly accounted for in the model using an additional volume fraction parameter, *f*_*w*_, as described in ref (42). The effect of the exchange of labile protons in the POPS headgroup and the peptide sequences had to be accounted for to properly analyze NR data obtained in different H/D-buffer mixtures. Proton–deuterium exchange in lipid headgroups was explicitly included in the modeling by modifying the scattering length of the PS headgroup using the lipid plugin provided by the Aurore software. During NR experiments, all peptides were prepared and injected in H_2_O, and contrast variation was applied by flushing the cells after the incubation. For this reason, we assumed that proton exchange was no longer possible if the peptides were inserted in the hydrophobic portion of the bilayer. It could still occur for those remaining on the surface or in the SLB portion accessible to solvent molecules. Therefore, an average SLD value for all peptides of 2.4 × 10^–6^ Å^–2^ was used to analyze NR data measured under multiple contrast conditions.

### 2.6 Circular Dichrosim

CD was used to characterize the secondary structure elements in the peptides. The peptides were dissolved in either 10 mM NaF, 20 mM Tris at pH 7.4, or TFE to a peptide concentration of 0.1 mg mL^–1^ for all variants. The samples in aqueous buffer were filtered (Low Protein Binding Durapore (PVDF) Membrane, 0.22 μm, Prod. No. SLGV012SL, Millex, Ireland) before measurement. Far-UV CD measurements were performed on a JASCO J-715 spectropolarimeter with a photomultiplier tube detector. Spectra were recorded every 1.0 nm in the range 185–260 nm. The temperature was kept at 20 °C, and measurements started after 5 min of equilibration. Subtraction of reference spectra (containing only buffer or TFE) was performed on all spectra. The ellipticity is reported as the mean residue molar ellipticity θ (deg·cm^2^·dmol^–1^) according to
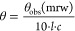
4where θ_obs_ is the ellipticity (deg), mrw is the mean residue molecular weight, *c* is the peptide concentration (g mL^–1^), and *l* is the optical path length of the cell (cm).

The obtained CD spectra were analyzed and fitted using three different methods: the online CD structure analysis tool DichroWeb^44^ with the CDSSTR algorithm using the SP175 data set, BeStSel^45,46^ (see http://bestsel.elte.hu/index.php), as well as SELCON3. The code for this function was originally made in Matlab in 2005 by the research group of Prof. B. A. Wallace, Birckbeck College, London. The MatLab code was updated in 2006–2007 to allow plotting and calculating the mean refitted spectrum to the query protein by S. V. Hoffmann, Aarhus University, Denmark. This code was adapted to Python by S. V. Hoffmann, Aarhus University, Denmark, in 2021, with the SP175 data set. These different methods gave information on the secondary structure elements present.

### 2.7 Synchrotron Radiation Circular Dichroism

SRCD was used in addition to CD to investigate the secondary structure of Hst5 in solution and the vicinity of spherical, non-porous silica nanoparticles (NPs, 150 nm diameter, Sigma-Aldrich) as well as PC_9_:PS_1_ lipid vesicles. Hst5 was dissolved in 10 mM NaF, 20 mM phosphate buffer, prepared by mixing sodium phosphate monobasic monohydrate and sodium phosphate dibasic dihydrate, at pH 7.4. The peptide was mixed with silica NPs at a 1:2 mass ratio (peptide:NPs) and a 1:1 mass ratio with the PC_9_:PS_1_ lipid vesicles. The measurements were performed at the AU-CD beamline at the synchrotron light source ASTRID2 (Department of Physics and Astronomy, Aarhus University, Denmark). Samples were measured in a 0.1 mm quartz cuvette at 20 °C. The spectra were recorded between 170 and 280 nm, but due to absorbance, only data in the range 178–280 nm were analyzed. The peptide concentration, in the order of 1 mg mL^–1^, was determined exactly from the obtained absorbance data during the CD measurement.

### 2.8 Small Angle X-ray Scattering

To gain information about the structure and aggregation behavior, SAXS measurements were performed at the European Synchrotron Radiation Facility (ESRF; Grenoble, France) using the BioSAXS beamline BM29.^47^ The peptide stock solutions were diluted to desired concentrations in series of approximately 0.5, 1, 2, and 5 mg mL^–1^, and the diluted samples were centrifuged at 14,000 rpm at room temperature for at least 30 min to remove potential large aggregates and/or impurities. The final concentration was determined using a Nanodrop 1000 instrument at 280 nm wavelength, and the analyte parameters, molecular weight of 3036 Da, and extinction coefficient of 2580 cm^–1^ M^–1^ were used. The SAXS data were obtained using an energy of 12.5 K e V and a sample-to-detector distance of 2.867 m resulting in a *Q* range of 0.0044–0.52 Å^–1^, where *Q* is still defined according to eq 2, where θ is now the scattering angle. In this case, *Q* does not depend on a specific direction in space. Samples were loaded into a flow-through quartz capillary using an autosampler robot (Arinax). Ten consecutive frames with an exposure time of 1 s each were recorded at 20 °C under flow to reduce radiation damage. The SAXS spectrum of the background, represented by the dialysis buffer, was measured before and after each sample’s acquisition, using the same exposure time as for the sample. The measurements were performed in replicates for the lowest concentrations, and final averages were determined in the data process. The forward scattering at *Q* = 0, *I*(0), was converted to absolute scale by measuring water scattering. The SAXS integration and initial processing used the BM29 automated pipeline.^48^ For the analysis of the data, the software Primus from the ATSAS package^49^ was utilized. The radius of gyration (*R*_g_) was determined for each sample by Gunier analysis in a *Q* range where the relation *Q* × *R*_g_ ≤ 1.1 held.

## 3 Results and Discussion

Hst5 and four variants thereof were investigated in three different environments: in bulk solution, in the vicinity of a negatively charged solid surface, and in the vicinity of a negatively charged SLB.

### 3.1 Conformational and Structural Properties in Solution

**Figure 2:**
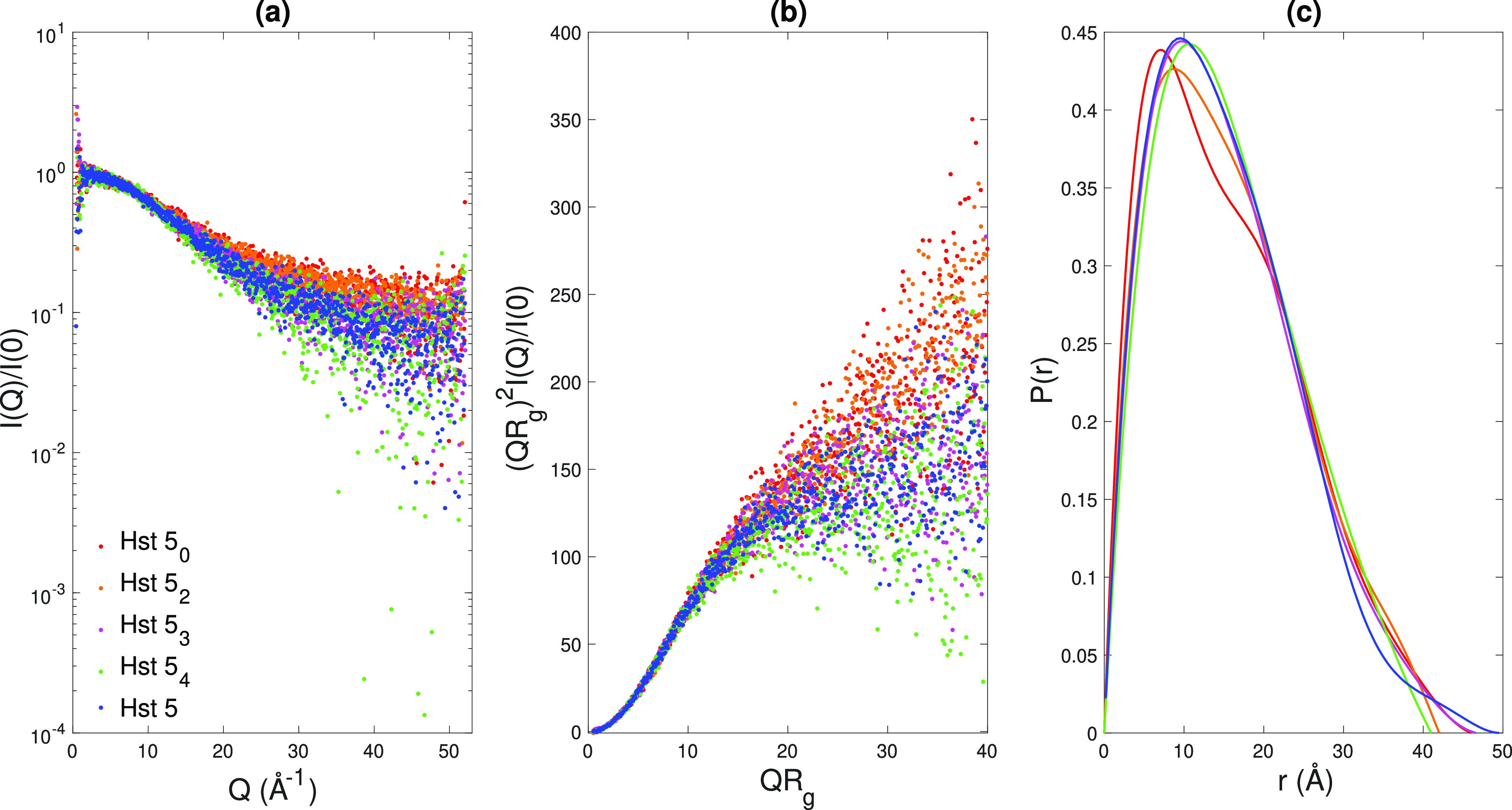
SAXS data for Hst5_0_ (red), Hst5_2_ (orange), Hst5_3_ (magenta), Hst5_4_ (green), and Hst5 (blue), displaying (a) an intensity curve, (b) a normalized Kratky plot, and (c) a pair distance distribution.

Normalized intensity curves, *I*(*Q*)/I(0) versus *Q*, Figure 2a, were obtained from SAXS measurements at an approximate peptide concentration of 1 mg mL^–1^; exact values are presented in Table 2. Normalized Kratky plots are depicted in Figure 2b, which transform the intensity curves by *R*_g_. The dimensionless Kratky plots give information about the shape of the peptides, where a bell-shaped Gaussian curve is indicative of a compact, globular peptide, whereas a plateau at high *Q* is characteristic of an unfolded and flexible peptide. A plateau is visible for all peptides, suggesting that Hst5 and the variants have an unfolded and flexible structure. However, there is a gradient of flexibility among the variants where Hst5_0_ (red) and Hst5_2_ (orange) are more flexible than Hst5 (blue), whereas Hst5_3_ (magenta) is very similar to WT Hst5, and Hst5_4_ (green) display a more globule like shape. The pair distribution functions, *P*(*r*)’s in Figure 2c, are a real space transform of the intensity curves using a reverse Fourier transform; they give information about the shape as well as the maximum dimension, *D*_max_, of the peptides. Here they show a broad distribution up to 50 Å, with a maximum just above 10 Å, which is roughly 50% of the maximum contour length (98.4 Å), calculated based on parameters previously used for Hst5.^13,50^ Since SAXS reports the conformational ensemble average, the peptide can attain quite compact conformations even though it is unfolded and flexible. The *R*_g_ was determined using the Guinier approximation and resulted in a *R*_g_ value of 11.6–12.0 Å for all peptides (see Table 2), which is slightly smaller than what has previously been reported for Hst5.^13^ It is, however, important to remember that these results are obtained at 10 mM NaCl, instead of the previously reported 140 mM NaCl, which may affect the intra- and intermolecular interactions in the system. However, the obtained results give information on the conformational ensemble under the same conditions as the other investigations in this study have been performed and are therefore relevant. *R*_g_ values for all the peptides indicate that the average sizes of the conformations they can attain are similar. However, as discussed above, the number of conformations available to them differs between the different variants, making them more or less flexible.

**Table 2:** Radius of Gyration (*R*_g_), Intensity at *Q* = 0 (*I*(0)), and *D*_max_ Values Determined for the Investigated Peptide Samples at 10 mM NaCl

Peptide	Concentration [mg mL^–1^]	*R*_g_ [Å]	*I*(0) [cm^–1^]	*D*_max_ [Å]
Hst5_0_	1.030	11.7 ± 0.4	2.49 ± 0.036	46.0
Hst5_2_	1.290	11.6 ± 0.3	3.37 ± 0.036	46.0
Hst5_3_	0.931	12.0 ± 0.4	3.22 ± 0.034	46.6
Hst5_4_	0.880	11.7 ± 0.4	2.31 ± 0.034	41.0
Hst5	1.463	11.8 ± 0.3	3.37 ± 0.035	49.5

The secondary structures of Hst5 and the variants were evaluated with CD spectroscopy, both in aqueous Tris buffer supplemented with 10 mM NaCl, as well as in TFE, representing a hydrophobic environment mimicking the hydrophobic region and thus the tails of a lipid bilayer. In addition to this, Hst5 was also investigated using SRCD to improve the S/N ratio at lower wavelengths, λ < 178; see Figure S2a. Despite the improved data quality, in the spectrum region where the main secondary structure elements can be evaluated, there are no apparent differences in the curves obtained using lab-based and synchrotron-based equipment. Hence, the former was routinely used to investigate differences in the secondary structure for all the variants.

**Figure 3:**
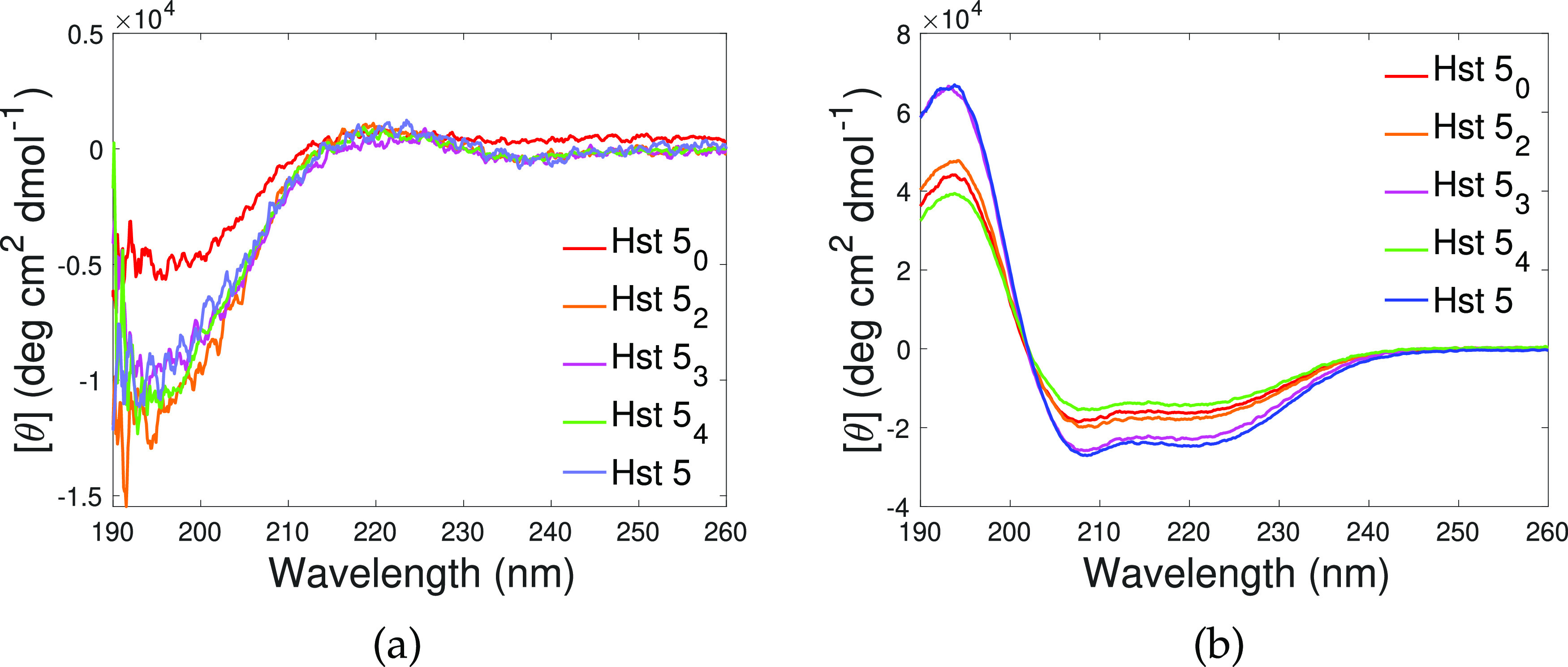
CD spectra obtained for Hst5 and variants thereof in (a) aqueous buffer and (b) TFE.

The curves obtained in the aqueous buffer are presented in Figure 3a, showing the ellipticity, θ, as a function of wavelength. Their shape indicates a mainly disordered secondary structure of the variants with a negative band near 195 nm and low values of θ above 210 nm.^51^ The data were analyzed using the BeStSel analysis, CDSSTR, and SELCON3. SELCON2 was used in cases where no SELCON3 solutions could be found. All methods predicted Hst5 and variants thereof to be mainly unordered, which includes structures determined as “unordered” or “others” by the methods, as well as “turns”. Hst5 and variants are also predicted to contain a substantial amount of β-structures, roughly 40%. Only a minor amount was predicted to be α-helical in the SELCON3/SELCON2 method; see Table S3. The secondary structures of Hst5 and variants were also investigated in TFE since previous studies^14,20^ have shown that Hst5 obtains an α-helical structure in non-aqueous solvents. An apparent increase in α-helical structure is observed when the peptides are dissolved in TFE, shown in Figure 3b by the strong negative bands at 208 and 222 nm.^51^ The data were analyzed using the same three methods as the data in the aqueous buffer, and it was found that α-helices were present to a larger extent in TFE than in the aqueous buffer, as presented in Table 3. All peptides are very similar in aqueous buffer, with almost the same structure in all cases, predicted by SELCON2/SELCON3, CDSSTR, and BeStSel, which shows that the predicted distribution between α-helix and β-sheets is quite similar. Hence, there are no indications that a change in secondary structure is induced upon the mutations of the peptide, and all peptides can be considered intrinsically disordered in aqueous solution and mainly α-helical in non-aqueous solution.

**Table 3:** Amount of the Different Secondary Structures of Hst5 and Variants in Aqueous Buffer (left)/TFE (right) Where the Value Is the Average of the Predicted Values from All Three Analysis Methods

Peptide	a-Helix (%)		b-Sheet (%)		Unordered (%)	
Hst5_0_	7	48	40	14	55	38
Hst5_2_	26	52	37	10	60	35
Hst5_3_	11	66	40	10	56	24
Hst5_4_	11	44	42	16	54	41
Hst5	7	65	41	7	56	27

### 3.2 Adsorption Properties and Structural Changes in the Vicinity of a Solid Surface

**Figure 4:**
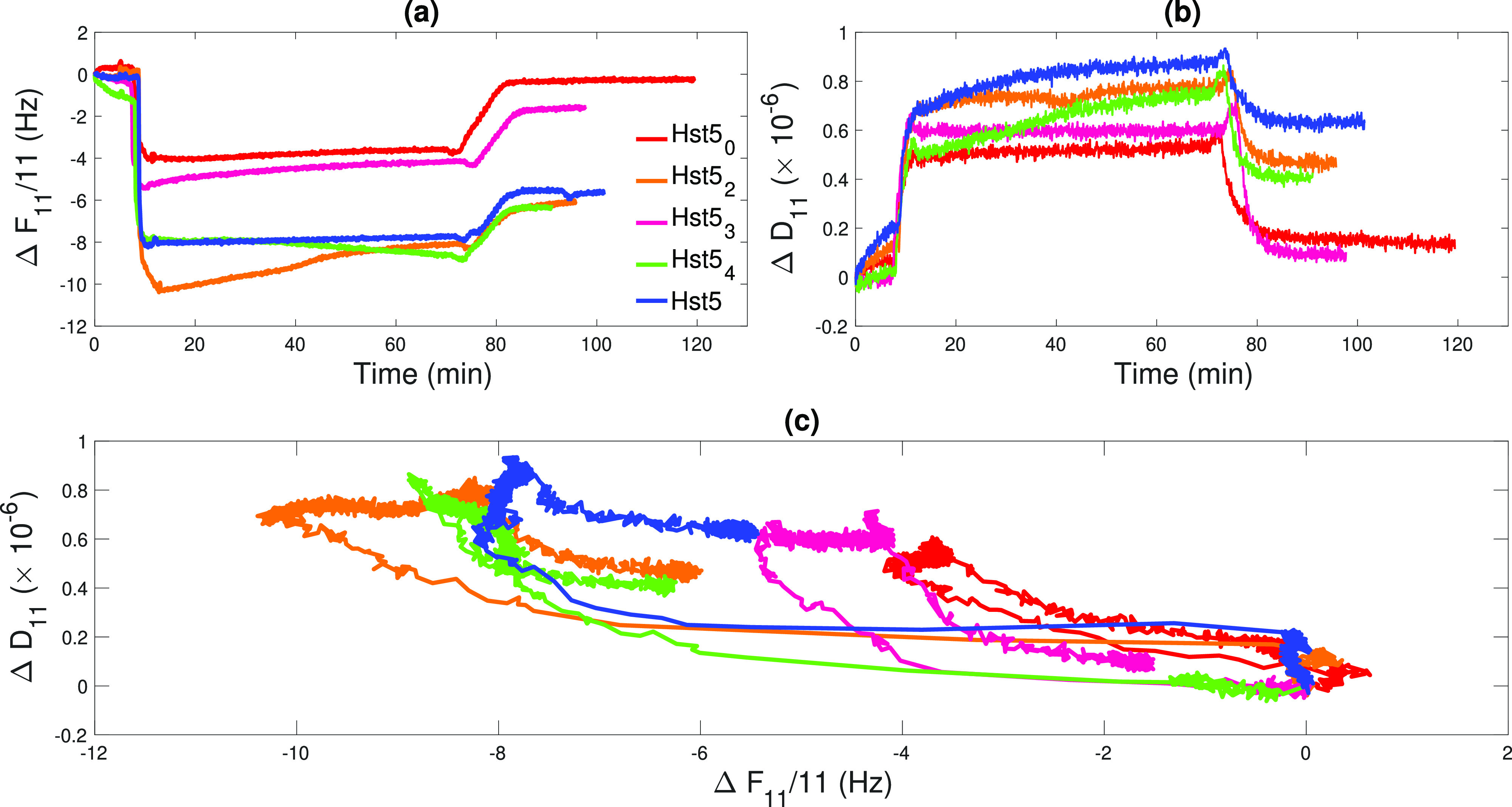
QCM-D results obtained for Hst5_0_ (red), Hst5_2_ (orange), Hst5_3_ (magenta), Hst5_4_ (green), and Hst5 (blue). (a) Normalized frequency shifts measured for the 11th overtone upon injection and incubation of the peptide solutions and rinsing with peptide-free buffer. (b) Change in the dissipation factor of the 11th overtone corresponding to the data shown in (a). (c) Dissipation vs frequency plot for the data reported in (a) and (b).

Information about the mechanical properties of the peptide films formed on the negatively charged silica surface was extracted from the interpretation of Δ*D*_*n*_ versus  plots as reported in ref (52). For all samples, the adsorption process was very fast, lasting less than 1 min as indicated by the rapid decrease of  visible at *t* ≈ 10 min in Figure 4a, where the  data are shown. For all samples, it was possible to quantify the adsorbed amount of the peptide by using eq 1, supported by the low shift in Δ*D*_11_, shown in Figure 4b. In particular, by using the values measured for the 11th overtone before rinsing (*t* ≈ 65 min, i.e., end of incubation) and after rinsing (*t* ≈ 90 min), the Δ*m* values reported in Table 4 were determined. It is worth noting that these values might also contain a contribution from coupled water molecules. Despite differences in the amount of adsorbed material, all samples followed a similar pathway of adsorption and desorption as indicated by the similar shape described by the dissipation plotted as a function of the corresponding normalized frequency shift as reported in Figure 4c. At the end of the incubation period, a similar shift in frequency is observed for Hst5_2_, Hst5_4_, and Hst5, approximately −8 Hz. For the Hst5_2_ sample an increase of  from *t* ≈ 15 min to *t* ≈ 65 min was shown. This could be explained by a change in the conformation of the peptide on the surface; the increase of the frequency could be originated by the transition of the peptide from a disordered to a more flat conformation, leading to a release of coupled water molecules. During the same period, the signal registered for the Hst5_0_ and Hst5_3_ samples remained constant, although it was limited to a smaller value of , indicating either a smaller peptide adsorbed amount or peptide adsorption in a different conformation compared to the other variants. Again, peptides adsorbed in a flatter conformation would result in smaller frequency shifts than if the peptide is extended out in solution, as the amount of coupled water would be smaller. Another possibility is that the peptides are adsorbed in a compact and collapsed random coil conformation, which would also give rise to a low shift in the monitored dissipation factor and a smaller frequency shift due to less water coupled to the peptides. It could also simply be due to a smaller amount of adsorbed peptide. However, the technique does not allow us to differentiate between these possible scenarios, and we can not rule out any of them with certainty. For all samples, a non-negligible fraction of peptide molecules was desorbed from the surface during the rinsing step, suggesting the presence of bound and loosely bound peptide molecules. A large removal of peptides was also observed for the two samples showing the lower adsorbed amount. Indeed, for Hst5_0_ and Hst5_3_, only 9% and 40% of the adsorbed peptides remained bound to the silica surface, respectively. These two peptides lack His residues at positions 3, 7, and 8, which is within the zinc motif suggested by Cragnell et al.^29^ in the zinc motif at positions 3, 7, and 8. In addition, they also lack the His residue at position 21. This suggests that the number of His and their position in the primary sequence is important for determining the strength of the interaction. From previous computational studies,^21,22^ it was found that, when Hst5 adsorbs to a negatively charged surface, the amino acids 5–13 are in the closest contact with the surface. Removing the His in this region, as in the case of Hst5_0_, and Hst5_3_, probably diminishes the attraction between this region and the surface, decreasing the adsorbed amount we observe from QCM-D. This, together with the discussion above, suggests that there is actually a smaller adsorbed amount for these two peptides (Hst5_0_ and Hst5_3_), rather than a difference in conformation, compared to the other peptides, upon adsorption. It is important to note that the net charge of the peptide is still +5; despite this, the variant with no His left in the sequence displays a much smaller shift in frequency, and upon rinsing, almost all of the adsorbed peptide is desorbed. Therefore, one can assume that the adsorption of Hst5 is not a simple electrostatic interaction between the surface and the peptide but rather an effect of the possible conformations the peptide can acquire upon interaction with the SiO_2_ surface. However, SRCD data of Hst5 near silica NPs indicate no change in the secondary structure, shown in Figure S2b. Aggregation of peptides and particles was apparent as the sample became milky during the measurements.

**Table 4:** Adsorbed Amount, Obtained by Values before (BR) and after Rinsing (AR) for Hst5 and Variants on a Bare SiO_2_ Surface Obtained with QCM-D^a^

Peptide	BR [ng cm^–2^]	AR [ng cm^–2^]
Hst5_0_	64	6
Hst5_2_	144	109
Hst5_3_	74	31
Hst5_4_	151	111
Hst5	138	99

a Based on the fluctuation of the QCM-D data, the relative uncertainty of the adsorbed amount values resulted in the range 0.5–1.0% for all data sets.

### 3.3 Penetration Depth of Peptides in a Negatively Charged SLB

**Figure 5:**
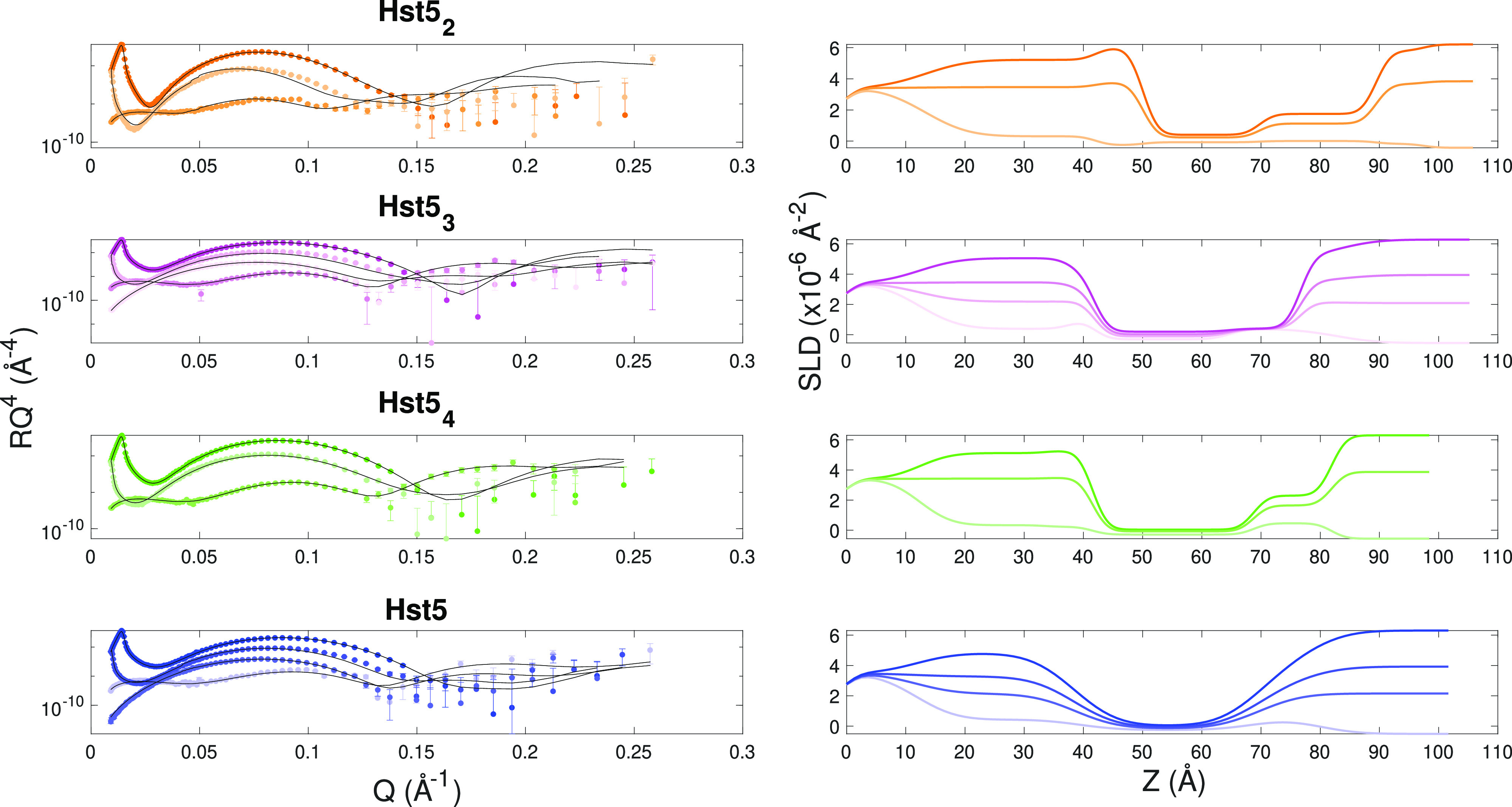
Left: Experimental reflectivity curves (points) and fit curves (lines) for Hst5_2_ (orange), Hst5_3_ (magenta), Hst5_4_ (green), and Hst5 (blue) samples collected in three contrasts (from light to dark colors: H-buffer, SiMB, 4MBuffer, and D-buffer). Right: SLD profiles corresponding to the model curves obtained from the analysis for the same samples reported on the left. The same color code used on the left applies.

**Figure 6:**
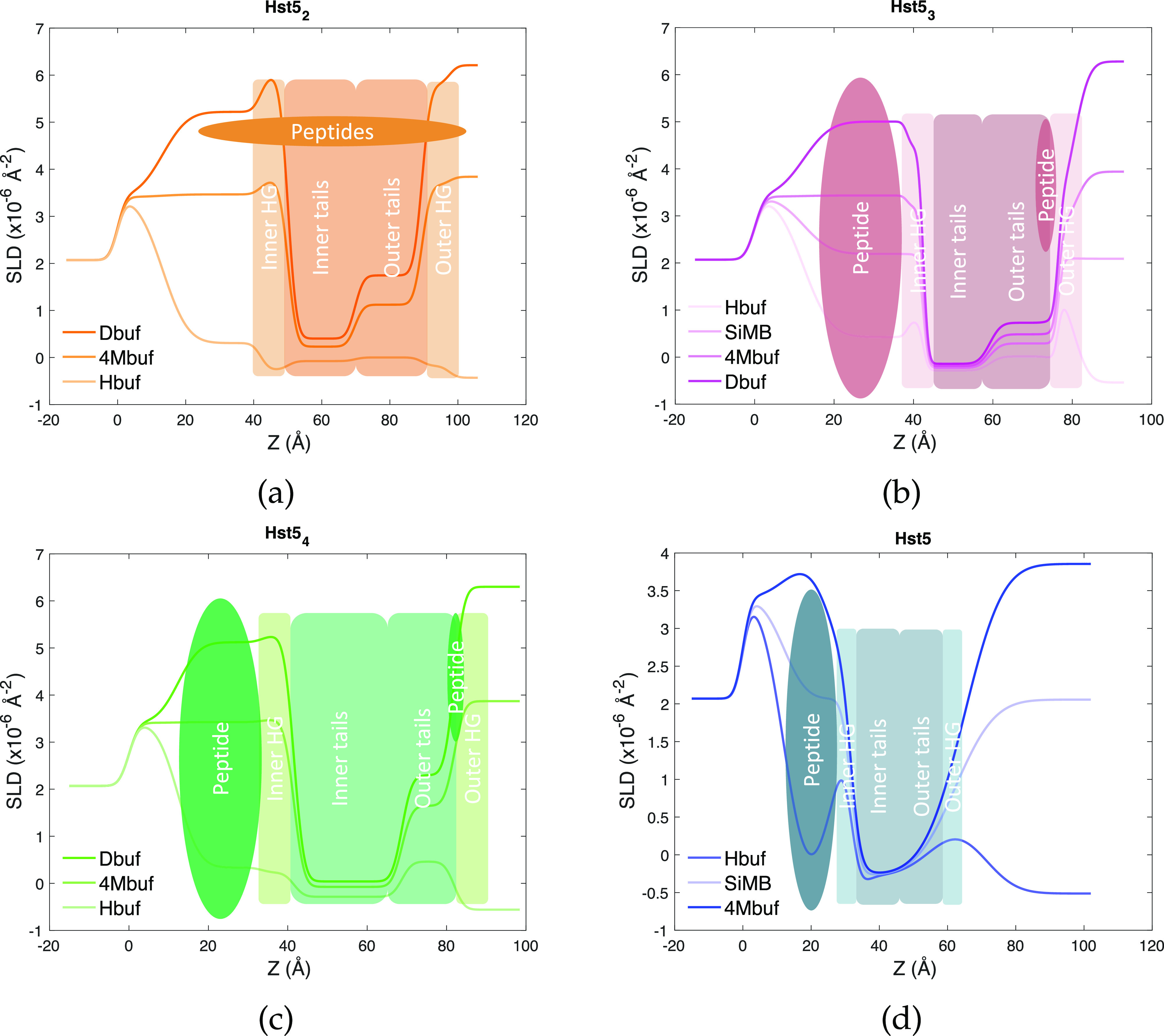
Schematic figures of the position of the peptide after rinsing from NR data.

In our previous paper,^30^ Hst5 was found to translocate across the bilayer without disrupting the internal structure and reside below the SLB on top of the solid silica surface. Therefore, we wish to investigate if this translocation was possible due to the number of His in the amino acid sequence. NR curves were measured under multiple contrast conditions after rinsing to determine the position of the peptides with respect to the SLB and the supporting surface and to detect structural changes induced by the peptides to the SLB. NR data, theoretical models, and the corresponding SLD curves obtained from the analysis are presented in Figure 5 for all samples except Hst5_0_ as the experimental data measured for this sample deviate from the above-described behavior. The Hst5_0_ data are shown in Figure S1 and discussed separately. For samples containing two or more His residues, the reflectivity curves could be modeled by only changing the thickness and SLD values previously obtained for the pristine SLBs, indicating the overall structure and molecular organization of the SLB were maintained. Such a behavior is similar to the one already observed for the WT peptide^30^ and confirmed in the present work. In particular, the peptide Hst5_4_ was found to reside within the headgroups of the outer leaflet and below the bilayer. Hst5_3_ was also found below the bilayer, within the headgroups of the inner leaflet and, additionally, within the tail and headgroup region of the SLB outer leaflet. Lastly, Hst5_2_ was located below the SLB and in all SLB regions. Moreover, depletion of lipids from the bilayer was detected in this sample, as indicated by the significant split of the SLD profiles in the outer leaflet region; see Figure 5. Pictorial sketches illustrating the location of the peptides with respect to the SLD profiles obtained from the modeling and with respect to the different SLB regions are given in Figure 6. Differences among the samples were also observed in the cushion thickness formed (see Table 5), showing a clear decrease in cushion thickness upon increasing the number of His. We suggest that this is due to the possibility of flatter adsorption when the number of His is higher.

**Table 5:** Thickness of the Cushion between the Solid Substrate and the SLB Formed upon Injection of the Peptide and Determined by the Modeling of NR Data

Peptide	Thickness [Å]
Hst5_2_	29 ± 3
Hst5_3_	26 ± 3
Hst5_4_	21 ± 2
Hst5	21 ± 1

It is worth noticing that the gap thickness for the Hst5 sample determined in the present work resulted in perfect agreement with the one reported previously,^30^ indicating the high experimental reproducibility of such a spontaneous process. These results clearly indicate that peptides with at least two His residues can translocate across a negatively charged SLB and accumulate between two like-charged surfaces, i.e., silica and SLB. The mechanism of the cushion formation could be explained by the fact that the His in the sequence can charge titrate, which would allow the charges of the peptide to be neutralized, enabling the peptide to traverse the bilayer without any pores or active transportation. It is worth noticing that, with the sensitivity of NR, it is not possible to exclude the formation of a few nanosized pores or defects, as the limit of detection is approximately set to 2–3% in volume fraction with the contrast scheme and instrumental configuration used. The hypothesis of the major role played by charge titration is supported by the fact that we observe an increased interaction with the bilayer when the number of His in the sequence is decreased, which could be explained by the fact that the peptide can no longer be charge titrated in the same fashion as the WT Hst5. In the case of the Hst5 variants, we also observe some alterations as defects and pores in the bilayer structure after interaction with the peptide. The above-described behavior does not apply to the peptide lacking all His residues, Hst5_0_. In this case, an important structural remodeling of the SLB was observed, as indicated by the radical changes in the reflectivity curves; see Figure S1. As is shown, the curves obtained after injection of Hst5_0_ no longer resemble that of a lipid bilayer; rather, they suggest forming a thick film on top of the solid substrate. By inspecting the raw data, we did not observe any trace of off-specular scattering nor Bragg peaks in the specular signal, suggesting the film’s absence of any particular order or periodicity. Attempts to model the curves collected using existing models did not succeed. Using the relation *d* = 2π/Δ*q*, a thickness of approximately 250 Å is obtained from the spacing between two subsequent fringes in the specular reflectivity data. The absence of signals indicating order or periodicity and the appearance of thick film fringes might suggest forming a mixed lipid–peptide film with an internal structure that NR cannot resolve or is not present.

As all of the peptides either cross or remain in the hydrophobic portion of the SLBs, the secondary structure of Hst5 upon adsorption to negatively charged SRCD characterized PC_9_:PS_1_ vesicles. No changes in the peptides’ secondary structure were observed for any of the investigated samples. This disagrees with results obtained in previous studies,^18,20^ where the vicinity of lipid vesicles induced a more α-helical structure compared to aqueous buffer.

### 3.4 Adsorption Strength and Processes

**Figure 7:**
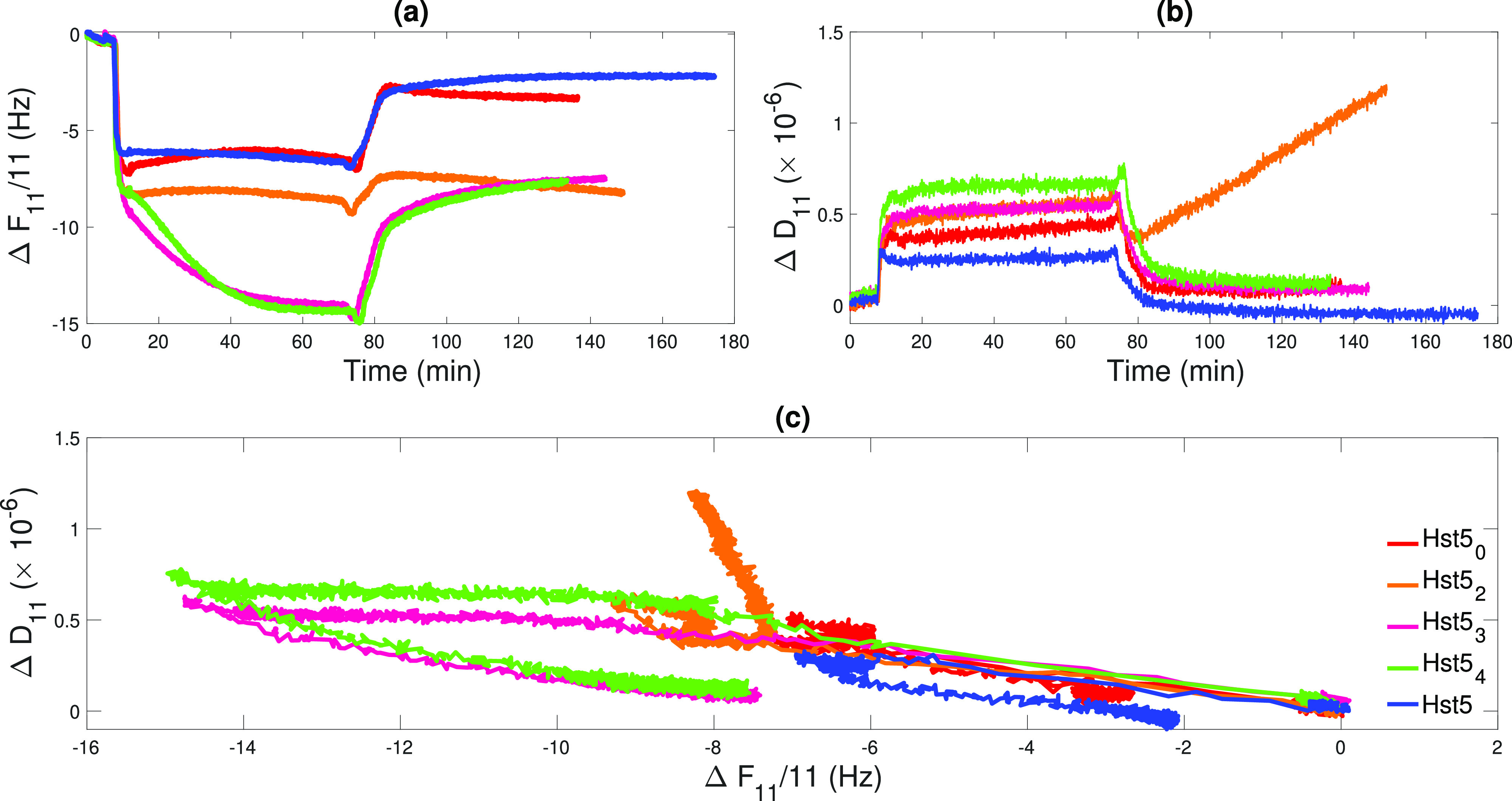
QCM-D data measured for Hst5_0_ (red), Hst5_2_ (orange), Hst5_3_ (magenta), Hst5_4_ (green), and Hst5 (blue) upon interaction with the SLB. (a) Normalized frequency shifts were measured for the 11th overtone during injection, incubation of the peptide solutions, and rinsing with peptide-free buffer. (b) Change in the dissipation of the 11th overtone corresponding to the data shown in (a). (c) Dissipation vs frequency plot for the data reported in (a) and (b). The initial baseline corresponds to the already deposited SLB.

QCM-D measurements were conducted for the same samples investigated by NR. As for NR, lipid bilayers were prepared by vesicle fusion on silica surfaces. Their quality was evaluated by the frequency shift and dissipation reached at the end of the formation process: if Δ*F*_*n*_/*n* ≈ 25 Hz and Δ*D*_*n*_ < 0.2 × 10^–6^, the SLBs were used for the peptide injection. Indeed, such values are expected for high-quality, defect-free SLBs composed of common phospholipid molecules.^53^ Even if most of the samples resulted in behaving as rigid thin films during most of the measurements (see the overlap in frequency and dissipation reported in Figure S10), we chose to interpret the QCM-D data by evaluating the trends of  and Δ*D*_*n*_ as a function of time and of Δ*D*_*n*_ as a function of . QCM-D data are reported in Figure 7, where only data measured for the 11th overtone are included for clarity. Data, including all measured overtones, are presented in Figure S10. For all samples, a fast adsorption process, similar to the one observed in the presence of a bare silica surface, is present after injecting the peptide solutions. This process is indicated by the rapid decrease in frequency and the corresponding increase of dissipation at *t* ≈ 10 min (panels a and b in Figure 7). The frequency shifts attained at the end of this fast adsorption are similar for different samples, ranging from −6 Hz (Hst5) to −9 Hz (Hst5_3_), corresponding to a net adsorbed amount between 106 and 160 ng cm^–2^ as determined by eq 1. These values are very similar to those obtained in the presence of the silica surface. Therefore, they could result from peptide adsorption on the surface of the SLB, a process mostly driven by electrostatic and hydrogen bonding. Differences between them can be induced either by a different amount of peptide or by more or less coupled water due to different orientations of the peptides at the surface.

For Hst5_0_, Hst5_2_, and Hst5, frequency and dissipation remained constant during peptide incubation (*t* ≈ 20–65 min), indicating that samples have reached equilibrium. In the case of Hst5_3_ and Hst5_4_, an additional decrease in frequency shift occurs during the entire incubation period, and it stops shortly before the rinsing step (*t* ≈ 65 min).

We suggest that this corresponds to either (i) a second, slow adsorption process, during which an additional peptide is added to the adsorbed layer without changing the dissipation of the layer, as indicated by the intermediate plateau in Figure 7c, or (ii) a slower translocation process than can keep the SLB structure rigid, i.e., no increase in dissipation while slowly lifting the bilayer from the silica surface leading to the increase of dynamically coupled water in the cushion region, resulting in the observed frequency decrease.

In the case of (i), the observed trend suggests that the adsorbing molecules are incorporated within the already existing peptide–lipid film without increasing the system roughness to a large extent or the peptides are adsorbing almost flat on the top of the SLB.

Interestingly, upon rinsing, the frequency returns to a value close to the one reached at the end of the fast adsorption process, suggesting that all the material adsorbed during the slow process is removed, in the case of (i), or that, in the case of (ii), the peptide molecules which have not entered the bilayer, i.e., the ones still on top of the bilayer, are removed. Despite this, the available data cannot discriminate between the described scenarios and the type of molecules removed from the sensor in the presence of lipids.

The absence of the second slow process for Hst5_0_, Hst5_2_, and Hst5 may indicate systems reaching a limit in adsorption extremely quickly, in the case of (i), or of a very fast translocation process, in the case of (ii). The rinsing step for these samples has a stronger effect as a large mass is removed. It is worth noting that, as determined by NR, the variants with zero and two His promoted a structural rearrangement of the SLB that, in the case of Hst5_0_, completely changed the SLB structure and, in the case of Hst5_2_, resulted in lipid depletion.

Although the QCM-D data do not directly support the results obtained by NR, an indication of different behavior of these peptides might be found in the peculiar behavior shown by the sample containing two His residues in positions 8 and 19. The rinsing step does not have the significant removal effect observed in NR experiments, as the frequency value remains almost constant between *t* = 65 min and *t* = 100 min. On the contrary, the dissipation increases dramatically for all measured overtones (see Figure S10), indicating significant changes in the viscoelastic properties of the sample. The peptide–lipid film might become softer, and such behavior, in the absence of changes in adsorbed mass, can indicate slow but lasting structural rearrangements; another possibility is that, upon a structural rearrangement, the amount of water dynamically coupled to the film increases making a quantitative determination of the adsorbed amount very difficult.^54^

## 4 Conclusions

Upon changing the number of His in the His-rich peptide Hst5, we observed different effects depending on the environment the peptides were exposed to, thus bulk condition and interaction with a solid surface such as silica or a lipid bilayer. Only minor conformational and structural differences were observed under bulk conditions upon varying the number of His in the peptide sequence. Hence, all peptides can be considered unordered and flexible in an aqueous buffer. No structural differences were observed near negatively charged silica NPs or PC_9_:PS_1_ vesicles. The adsorption profiles obtained upon adsorption to a bare silica surface do not differ between the different variants. However, the adsorbed amount does. The two variants lacking His residues in the N-terminal, Hst5_0_ and Hst5_3_, display a significantly lower adsorbed amount than the rest of the investigated peptides. This region has previously been shown in computational studies as part of the sequence in the closest contact with the surface. We suggest that the smaller adsorbed amount for these two variants is either due to an actual smaller amount of peptides adsorbed or due to the fact that they are adsorbed in a more compact and collapsed random coil conformation, to which less water would be coupled, leading to a smaller shift in frequency. These results show that the silica surface in all surface experiments does not induce any differences in the adsorption profile. However, it does affect the adsorbed amount in two of the peptides, where the position of the His seems to be the determining factor.

Upon adsorption to an SLB, a similar adsorption profile as to bare silica is observed for Hst5_0_ and Hst5_2_, and Hst5. However, for Hst5_3_ and Hst5_4_ an additional decrease in the frequency shift is observed upon incubation, and we suggest that this is due to either an additional, slower adsorption process where the additional peptide is added to the layer or a slower translocation across the bilayer where the decrease in frequency shift then would correspond to an increase in coupled water below the bilayer.

In addition to the appearance of a slow and reversible adsorption process, the location and the ability of the peptides to penetrate the SLB were influenced by the number and position of His residues in the sequence. NR indicated that the penetration depth and capability to translocate across the lipid bilayer increase as the number of His in the peptide sequence increases. Moreover, peptides containing zero or two His might induce a destructing and remodeling of preexisting SLBs, suggesting a different interaction mechanism than the other variants investigated in this work.

We can therefore conclude that the His could, in fact, be important for the effect of the AMP since it, in this case, affects the translocation properties, which is vital for the peptide to be effective. Our proposed hypothesis regarding the mechanism is supported by the obtained results, where the ability to charge titrate with a high percentage of His in the sequence drives the peptide through the bilayer and into the solid surface. However, the route to get there has been proven not only to depend on the number of His in the sequence but also on their position relative to one another.

## Supporting Information Available

Analysis of additional NR data, additional overtones of QCM-D data, SRCD data, additional CD fits, and additional SAXS data (PDF)
