# Supplementary material for: Interaction of a Histidine-Rich Antimicrobial Saliva Peptide with Model Cell Membranes: The Role of Histidines
Source: Langmuir. 2023 May 25;39(22):7694–706. doi: 10.1021/acs.langmuir.3c00498 (PMC10249418; doi:10.1021/acs.langmuir.3c00498)
Supplement: Supplementary file 1 — la3c00498_si_001.pdf [file la3c00498_si_001.pdf]

Supporting information: Interaction of a histidine-rich antimicrobial saliva peptide with model cell membranes: The role of histidines.

Amanda E. Skog,[†] Giacomo Corucci,[‡] Mark D. Tully,[¶] Giovanna Fragneto,[§,∥] Yuri Gerelli,[*,⊥,#] and Marie Skepö[*,†,@]

†*Division of Theoretical Chemistry, Department of Chemistry, Lund University, P.O. Box 124, SE-221 00, Lund, Sweden*

‡*Institut Laue-Langevin, Partnership for Soft Condensed Matter, 71 avenue des Martyrs, 38000, Grenoble, France*

¶*BM29 BIOSAXS, European Synchroton Radiation Facility, 71 avenue des Martyrs, Grenoble, Isère 38043, France*

§*Institut Laue-Langevin, 71 avenue des Martyrs, 38000, Grenoble, France*

∥*European Spallation Source ERIC, P.O. Box 176, SE-221 00 Lund, Sweden*

⊥*CNR Institute for Complex Systems, Uos Sapienza, Piazzale Aldo Moro 2, 00185 Roma, Italy*

#*Department of Physics, Sapienza University of Rome, Piazzale Aldo Moro 2, 00185 Roma, Italy*

@*LINXS - Institute of Advanced Neutron and X-ray Science, Scheelevägen 19, SE-233 70, Lund, Sweden*

E-mail: yuri.gerelli@roma1.infn.it; marie.skepo@teokem.lu.se

S1 NR data

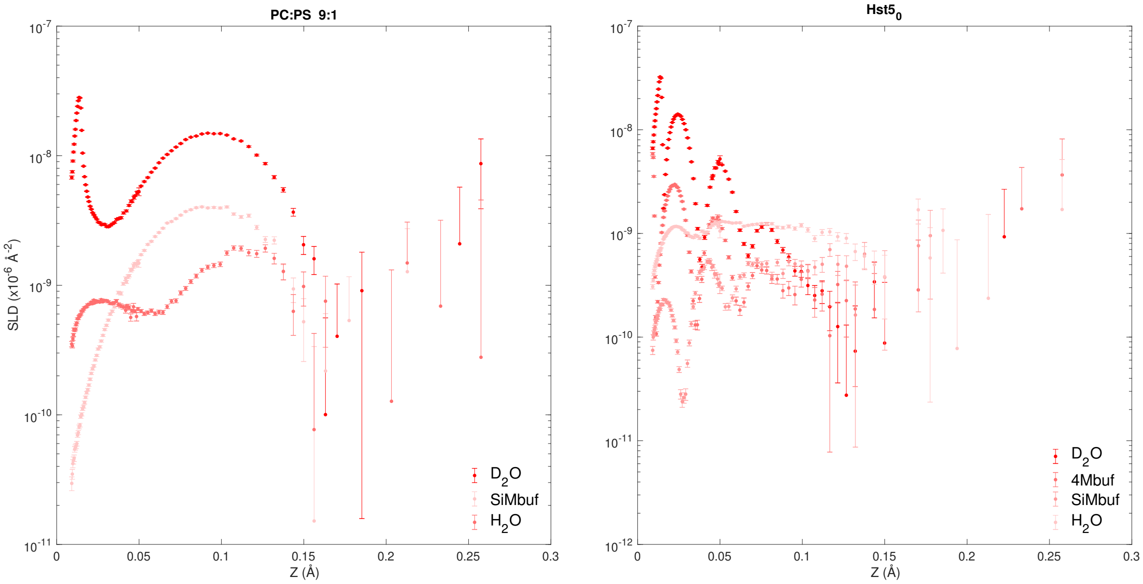

Figure S1: Reflectivity curves for a pristine PC_9:PS_1 bilayer measured with contrast variation as indicated in the legend (left) and reflectivity curves for the same bilayer upon interaction with $Hst5_0$ (right).

S2 SRCD

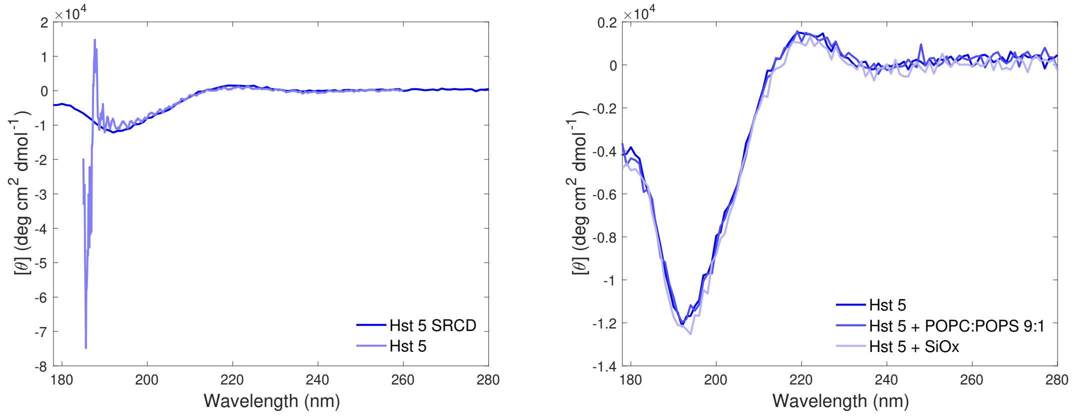

(a) (b)

Figure S2: CD curve in aqueous buffer (a) and place holder for DichroWeb analysis (b). CD curve in TFE (c) and place holder for DichroWeb analysis (d).

S3 CD fitting

Table S1: Amount of the different secondary structures of the peptides in aqueous buffer predicted by 1) SELCON3, *SELCON2, 2) CDSSTR 3) BeStSel

	α-helix (%)			β-sheet (%)			Unordered (%)		
$Hst5_0$	7*	0	0	45*	37	39	47*	58	61
$Hst5_2$	26*	0	0	26*	45	39	67*	53	61
$Hst5_3$	11*	0	0	35*	43	43	56*	55	57
$Hst5_4$	11*	0	0	34*	46	47	56*	53	54
Hst5	7	6	0	42	43	37	54	50	63

Table S2: Amount of the different secondary structures of the peptides in TFE predicted by 1) SELCON3, *SELCON2, 2) CDSSTR 3) BeStSel

	α-helix (%)			β-sheet (%)			Unordered (%)		
$Hst5_0$ TFE	49	47	49	16	20	5	34	34	46
$Hst5_2$ TFE	52	48	56	6	18	5	32	33	40
$Hst5_3$ TFE	67	53	77	10	15	5	22	32	18
$Hst5_4$ TFE	46	44	42	17	20	10	37	36	49
Hst5 TFE	70	55	70	7	13	2	23	31	28

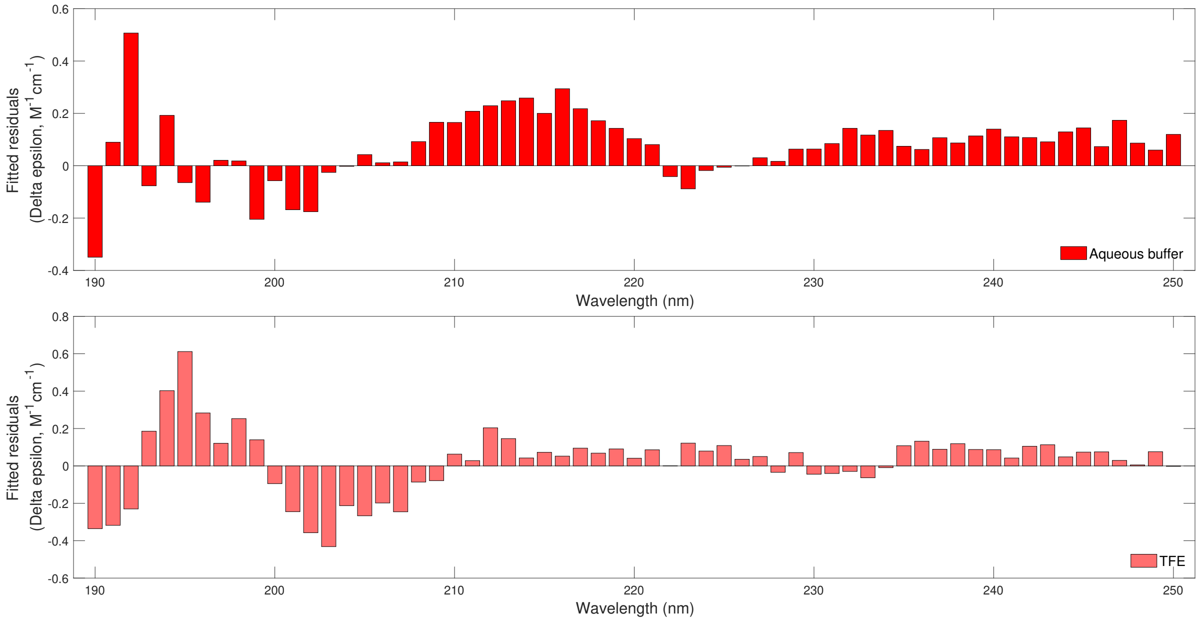

Figure S3: Fitted residuals using BeStSel for CD spectra of $Hst5_0$ in 10 mM NaCl, 20 mM TRIS at pH 7.4 (top) and TFE (bottom). The CD curves are graphically displayed in Figure 3 in the paper.

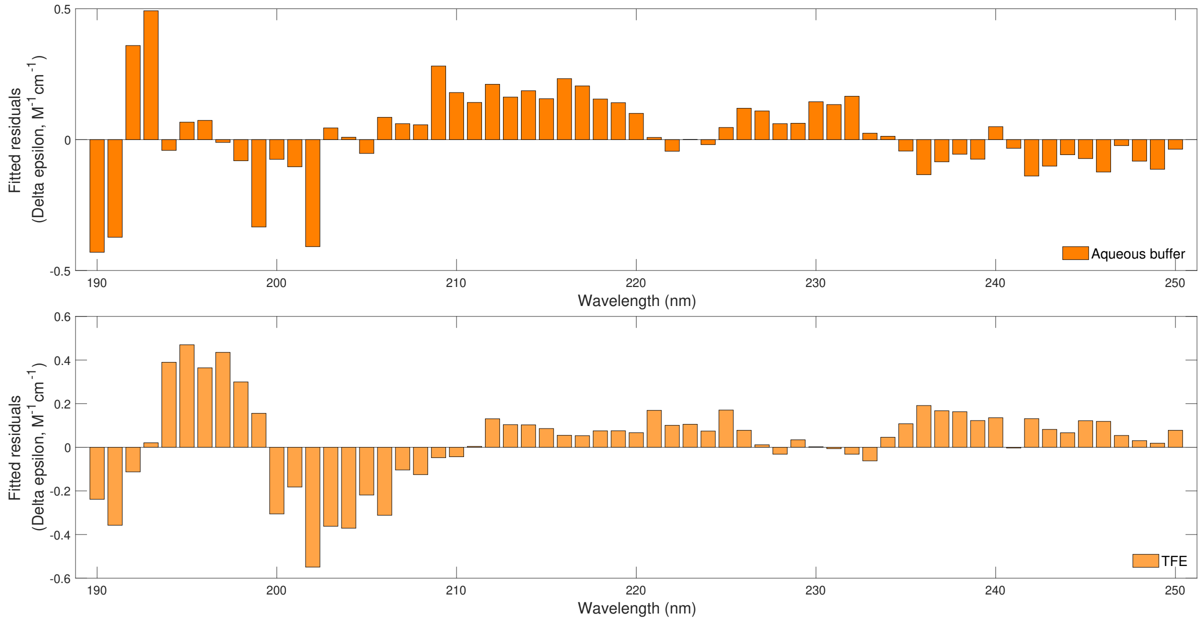

Figure S4: Fitted residuals using BeStSel for CD spectra of $Hst5_2$ in 10 mM NaCl, 20 mM TRIS at pH 7.4 (top) and TFE (bottom). The CD curves are graphically displayed in Figure 3 in the paper.

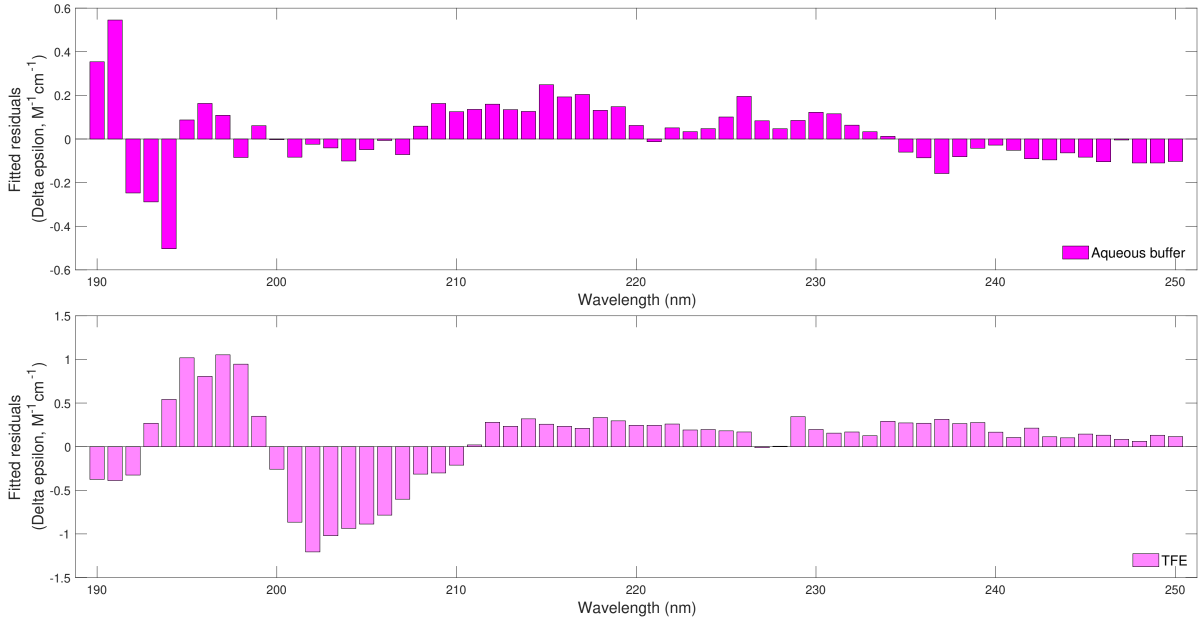

Figure S5: Fitted residuals using BeStSel for CD spectra of $Hst5_3$ in 10 mM NaCl, 20 mM TRIS at pH 7.4 (top) and TFE (bottom). The CD curves are graphically displayed in Figure 3 in the paper.

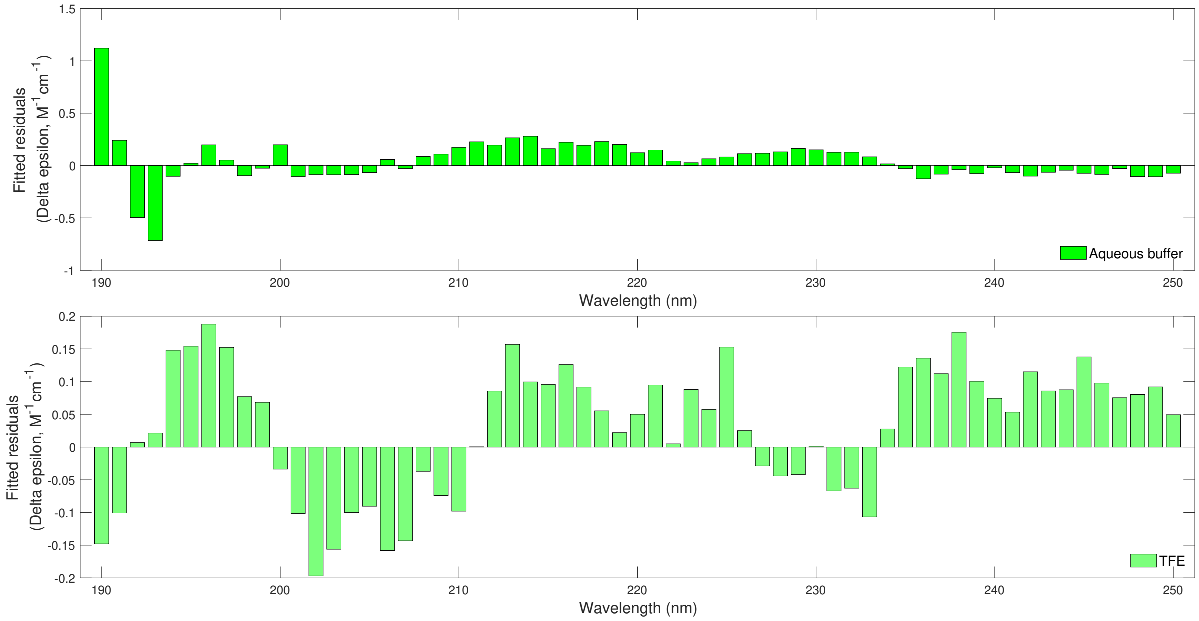

Figure S6: Fitted residuals using BeStSel for CD spectra of $Hst5_4$ in 10 mM NaCl, 20 mM TRIS at pH 7.4 (top) and TFE (bottom). The CD curves are graphically displayed in Figure 3 in the paper.

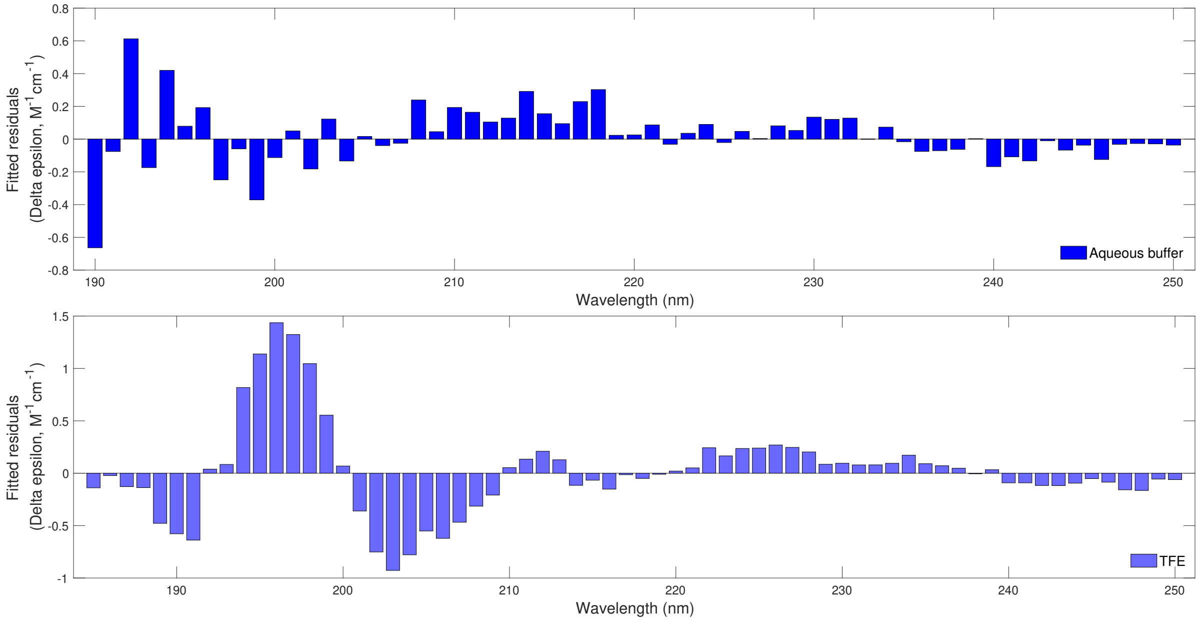

Figure S7: Fitted residuals using BeStSel for CD spectra of Hst5 in 10 mM NaCl, 20 mM TRIS at pH 7.4 (top) and TFE (bottom). The CD curves are graphically displayed in Figure 3 in the paper.

Table S3: Error estimates obtained from the different methods used to fit the CD data in aqueous buffer.

	SELCON3, *SELCON2		CDSSTR		BeStSel	
	# fits	RMSD	# fits	NRMSD	RMSD	NRMSD
$Hst5_0$	70*	0.0210	2	0.255	0.1493	0.07069
$Hst5_2$	52*	0.0279	12	0.148	0.1650	0.03980
$Hst5_3$	20*	0.0200	10	0.147	0.1535	0.04391
$Hst5_4$	70*	0.0131	9	0.055	0.2213	0.05851
Hst5	12	0.0129	4	0.020	0.1790	0.04475

Table S4: Error estimates obtained from the different methods used to fit the CD data in TFE.

	SELCON3		CDSSTR		BeStSel	
	# fits	RMSD	# fits	NRMSD	RMSD	NRMSD
$Hst5_0$ TFE	18	0.0916	65	0.005	0.1765	0.00937
$Hst5_2$ TFE	23	0.1215	62	0.006	0.1921	0.00943
$Hst5_3$ TFE	30	0.1237	35	0.004	0.4541	0.01632
$Hst5_4$ TFE	10	0.0119	63	0.008	0.1014	0.00614
Hst5 TFE	21	0.0900	26	0.004	0.4289	0.01579

S4 SAXS data

Table S5: Rg and I(0) values for Hst5 and variants at 150mM NaCl.

Peptide	Concentration ($mg\,mL^{-1}$)	R_g [Å]	I(0) [cm^{-1}]
$Hst5_0$	0.830	12.2 ± 0.4	2.26 ± 0.038
$Hst5_3$	0.931	13.0 ± 0.4	3.25 ± 0.045
$Hst5_4$	1.330	12.9 ± 0.2	3.70 ± 0.034
Hst5	0.853	11.4 ± 0.5	2.14 ± 0.045

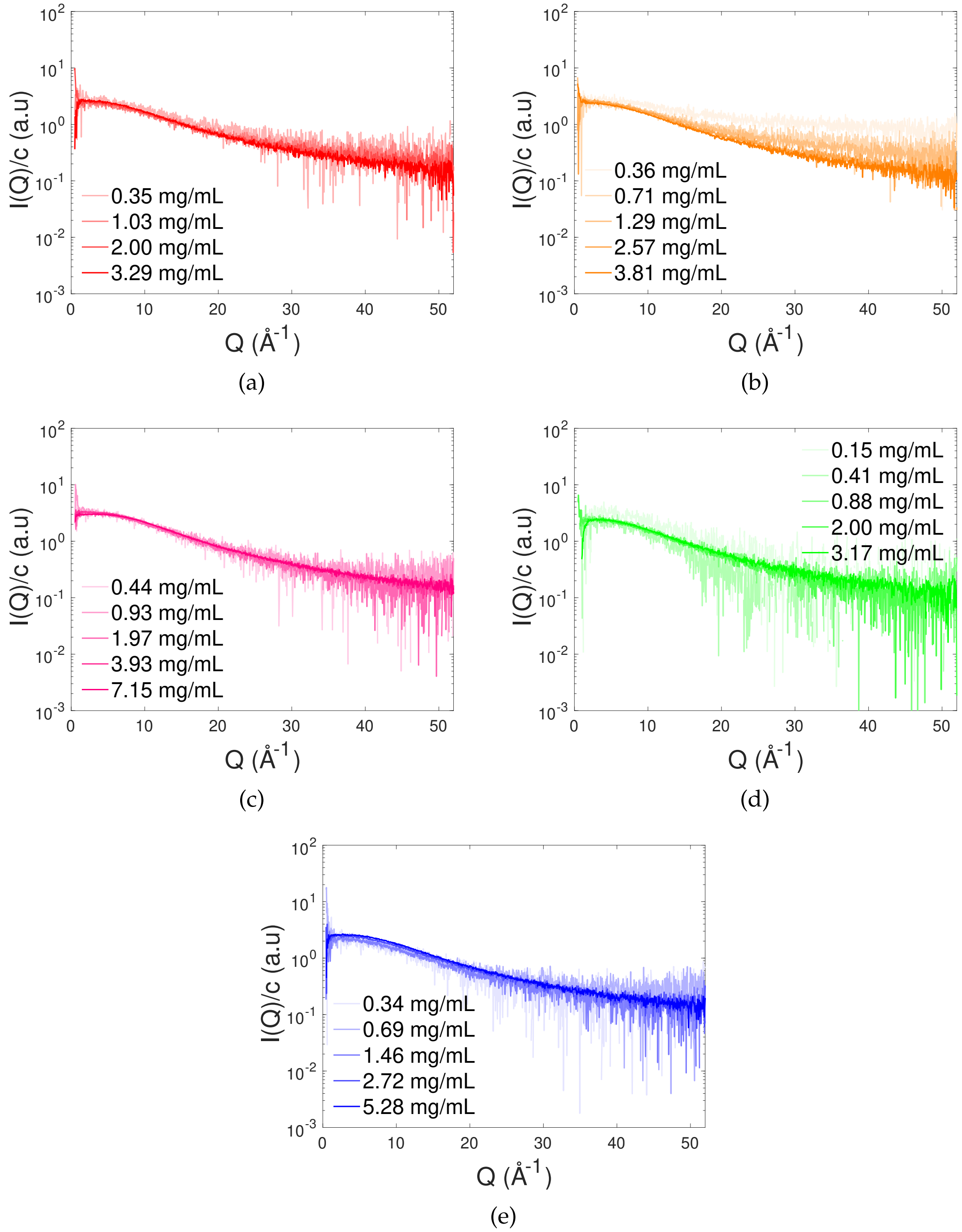

Figure S8: Intensity curves obtained by SAXS at different concentrations for (a) $Hst5_0$, (b) $Hst5_2$, (c) $Hst5_3$, (d) $Hst5_4$, (e) Hst5 at 10 mM NaCl.

S5 QCM-D data

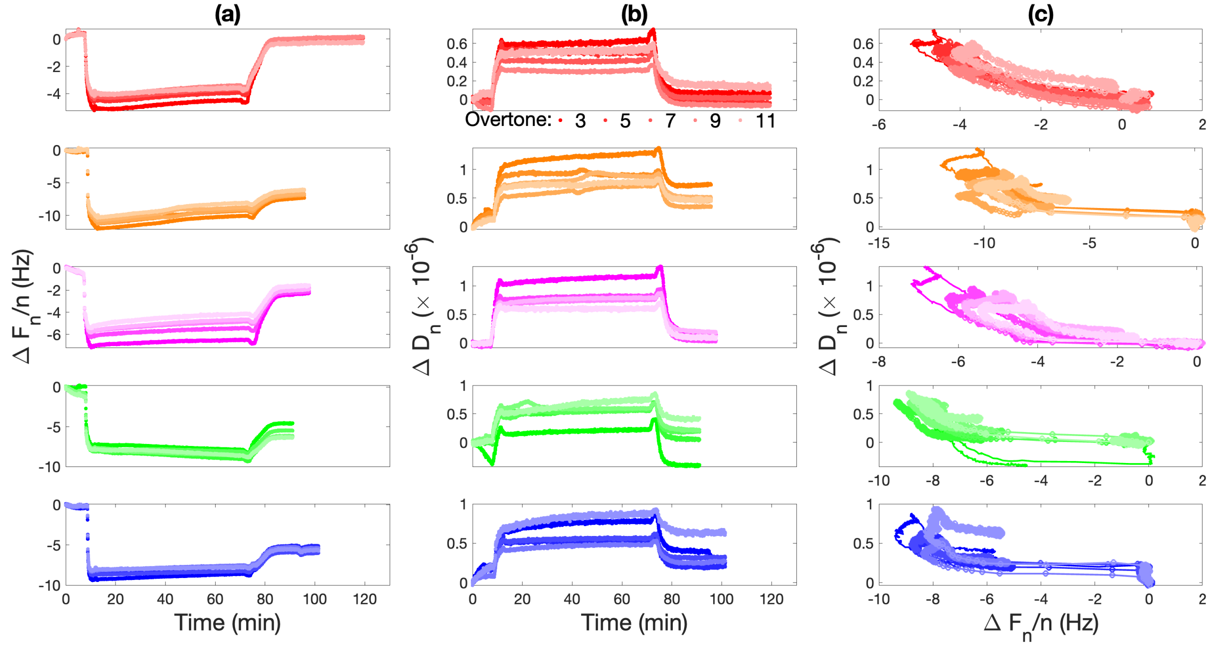

Figure S9: QCM-D results obtained for $Hst5_0$ (red), $Hst5_2$ (orange), $Hst5_3$ (magenta), $Hst5_4$ (green), and Hst5 (blue) where (a) displays the normalised frequency shifts upon addition of peptide, (b) the change in dissipation, and (c) the variation of dissipation as a function of the corresponding frequency shift. In all graphs, all measured overtones (from 3 to 11) are shown.

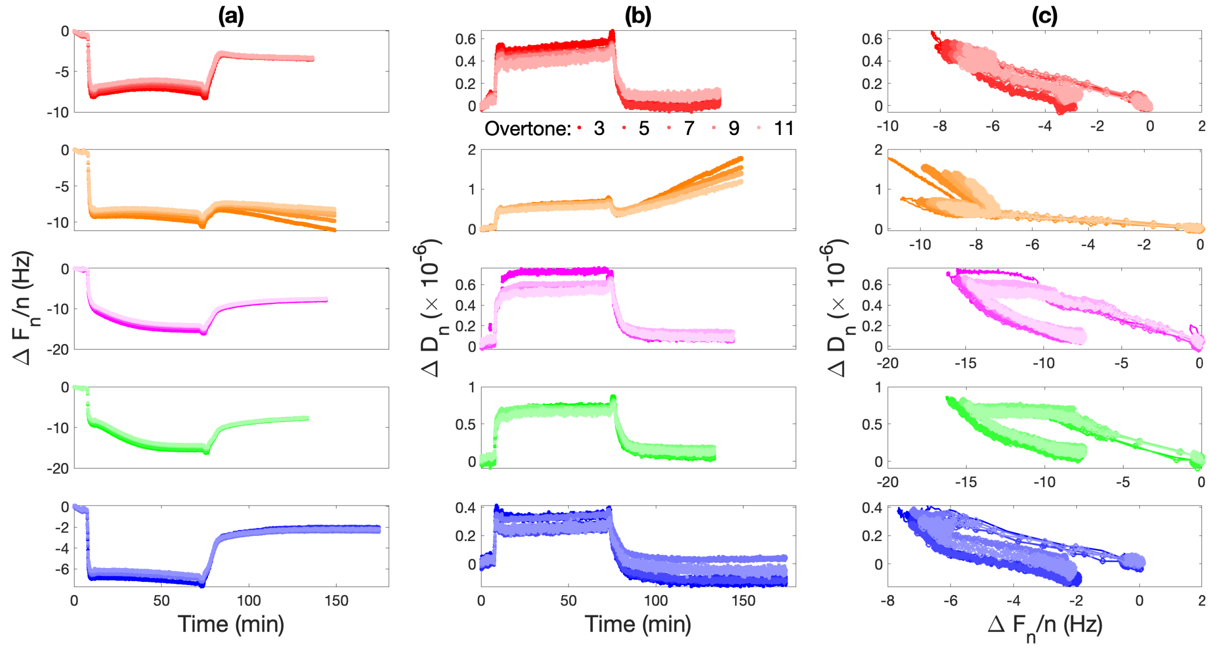

Figure S10: QCM-D results obtained for $Hst5_0$ (red), $Hst5_2$ (orange), $Hst5_3$ (magenta), $Hst5_4$ (green), and Hst5 (blue) where (a) displays the normalised frequency shifts upon addition of peptide, (b) is the change in dissipation, and (c) is the variation of dissipation as a function of the corresponding frequency shift. In all graphs, all measured overtones (from 3 to 11) are shown. Notice that the bilayer is already deposited and that a zero frequency here corresponds to the SLB.
